# Intrahepatic cholestasis of pregnancy after ovarian hyperstimulation syndrome with wild-type ABCB4 gene: a peculiar case and literature review

**DOI:** 10.1186/s12905-023-02471-4

**Published:** 2023-06-17

**Authors:** Loris Marin, Guido Ambrosini, Ludovica Nuzzi, Giovanni Buzzaccarini, Federica Esposito, Giampiero Capobianco, Vito Chiantera, Antonio Simone Laganà, Alessandra Andrisani

**Affiliations:** 1 Department of Women’s and Children’s Health, University of Padua, Padua, Italy; 2 Obstetrics and Gynaecology Unit, IRCCS San Raffaele Scientific Institute, Vita-Salute San Raffaele University, Milan, Italy; 3 Department of Surgical, Microsurgical and Medical Sciences, Gynecologic and Obstetric Clinic, University of Sassari, Sassari, Italy; 4 Unit of Gynecologic Oncology, ARNAS “Civico – Di Cristina – Benfratelli”, Department of Health Promotion, Mother and Child Care, Internal Medicine and Medical Specialties (PROMISE), University of Palermo, Piazza Nicola Leotta 4, 90127 Palermo, Italy

**Keywords:** Intrahepatic cholestasis of pregnancy, First trimester, Ovarian hyperstimulation syndrome, In vitro fertilization, Genetic polymorphisms

## Abstract

**Background:**

Intrahepatic cholestasis of pregnancy (ICP) in the first trimester occurring after ovarian hyperstimulation syndrome (OHSS) is a rare condition and few cases are reported in the literature. Hyperestrogenism may explain this problem in genetically predisposed women. The objective of this article is to report one of these rare cases and offer an overview of the other published cases.

**Case presentation:**

We report a case of severe OHSS followed by ICP in the first trimester. The patient was admitted to the intensive care unit and was treated according to the guidelines for the management of OHSS. Moreover, the patient also received ursodeoxycholic acid for ICP, which brought to an improvement of her clinical conditions. The pregnancy continued without other complications until the 36^th^ week of gestation, when the patient developed ICP in the third trimester and underwent cesarean section for increased bile acid levels and cardiotocographic (CTG) pathologic alterations. The newborn was a healthy baby weighing 2500 gr. We also reviewed other case reports published by other authors about this clinical condition. We present what is, to our knowledge, the first case of ICP developed in the first trimester of pregnancy after OHSS in which genetic polymorphisms of *ABCB4 (MDR3)* have been investigated.

**Conclusions:**

ICP in the first trimester might be induced by elevated serum estrogen levels after OHSS in genetically predisposed women. In these women, it might be useful to check for genetic polymorphisms to know if they have a predisposition for ICP recurrence in the third trimester of pregnancy.

## Background

Intrahepatic cholestasis (ICP) is the most common liver disorder to occur during pregnancy. However, it is a relatively rare condition with an incidence of 0.1%-2% among all pregnancies and it is characterized by generalized itch and liver function abnormalities in patients without other underlying conditions [1]. It typically occurs during the third trimester, although cases of jaundice or cholestasis have been reported also during the first trimester both in spontaneous pregnancies [2], as well as in pregnancies obtained with assisted reproduction technologies (ART) and complicated by ovarian hyperstimulation syndrome (OHSS) [3]. OHSS is the most serious complication of controlled ovarian hyperstimulation (COH) and it is characterized by a broad spectrum of signs and symptoms that include abdominal distention and discomfort, enlarged ovaries, ascites, and other complications associated with enhanced vascular permeability [4]. The incidence of moderate OHSS is estimated to be about 3–6%, while the severe form is relatively rare, with an incidence of 0,1–3% among all in vitro fertilization (IVF) cycles [5]. Main risk factors for developing OHSS are young age, lean body mass and polycystic ovary syndrome [4, 5]. Moreover, attention should be given to the gonadotropin administration during the COH, since different drugs and dosages are available [6]. Indeed, infertility pathways relies on the management of different causes and conditions [7, 8], and the choice of addressing the patient to ART should be carefully balanced in the light of available evidence in a shared decision-making environment with the patient [9].

In this report, we describe the case of a patient with a critical OHSS who got pregnant and developed ICP during the first trimester.

## Case presentation

The patient was a 34-year-old nulligravid Caucasian woman, with a history of infertility due to bilateral tubal occlusion and oligoovulation. She underwent an in vitro fertilization (IVF) cycle with Gonadotropin Releasing Hormone agonist (GnRH-a) long protocol. After down-regulation with leuprolide acetate, COH was performed using recombinant Follicle-Stimulating Hormone (rFSH) (Gonal F®, Merck Serono) with a daily dose of 225 IU for eleven days. Ovulation induction was obtained through recombinant human Chorionic Gonadotrophin (rhCG) (250 mcg s.c., Ovitrelle®, Merck Serono) administration when three follicles reached 17 mm diameter. On the trigger day, the level of estradiol was 27,496 pmol/L. Ultrasound-guided transvaginal follicular aspiration was performed 35 h after rhCG administration and eight mature oocytes were retrieved. The same day a biochemical evaluation of hematocrit, coagulation, serum albumin, liver and renal functions was performed, and all results were within the normal range.

The woman was asymptomatic, and two day-3 embryos were transferred. The luteal phase was supported with vaginal progesterone at a dose of 200 mg, twice a day (Prometrium®, Rottapharm). Twelve days after embryo transfer, the patient began to develop nausea and vomiting, abdominal pain, dyspnea and oliguria. Physical examination revealed paleness, dehydration, abdominal tension and lung hypoventilation. The patient was apyretic and had normal blood pressure, pulse rate and oxygen saturation values. The patient’s weight was 86.5 kg at the first control. Transvaginal sonography demonstrated an ovarian enlargement (left ovary 84 × 63 mm, right ovary 79 × 61 mm) and massive ascites. Blood analysis indicated hemoconcentration (hematocrit 46,1%, white blood cells 19.010/ml, platelets 855.000/ml), hyperkalemia (5,1 mmol/l) and hypoproteinemia (total protein concentration 59 g/l and albumin 27 g/l). There was no evidence of renal and hepatic failure. Serum b-hCG was 182 U/L. The patient was admitted to the Hospital with a diagnosis of critical OHSS [5] and underwent continuous monitoring and a supportive therapy consisting of oral and intravenous hydration, colloid fluids, crystalloid fluids, human albumin infusion and prophylactic doses of low molecular weight heparin. Daily records of fluid intake and urine output were performed. Despite a conservative approach, the clinical condition did not improve, the intensity of dyspnea and abdominal tension increased, and the patient’s weight reached 87,5 kg. After 4 days of treatment the patient complained of dyspnea, tachycardia and sweating, and was therefore treated also with oxygen, furosemide (10 mg/day) and cabergoline (0,5 mg/day) and underwent abdominal paracentesis. The patient was transferred to the Intensive Care Unit (ICU) of the Hospital because of the onset of mild hydropericardium and moderate pleural effusion. The cabergolin therapy was not tolerated by the patient, due to severe asthenia, and was therefore interrupted. During the recovery in ICU, the patient was treated with parental nutrition, continuous dopamine infusion (3 *γ*/kg/min), intravenous hydration and plasma expanders; paracentesis was repeated, and an albumin therapy was started for ascites and mild pleural and pericardiac effusion. When the respiratory and abdominal symptoms worsened, beta-hCG level was 1057 U/L. At that time, the ultrasound scan showed a single gestational sac with a fetus at the 5^th^ week of gestation. Laboratory tests revealed a reduction in hemoconcentration (hematocrit 41,5%, white blood cells 13.000/ml, platelets 385.000/ml), but an increase of aspartate aminotransferase (AST) and alanine aminotransferase (ALT), which were 0,67 ukat/l and 1,17 ukat/l, respectively. Over the next 15 days, a gradual reduction of ovarian size and ascites, as well as of the pleural and pericardial effusions, were achieved and the patient was transferred to the Obstetric Unit. A new transvaginal ultrasound exam revealed two gestational sacs, one containing a fetus at 6 weeks of gestational age, while the other one was a blighted ovum; in addition, ultrasound scan revealed a reduced ascites and ovarian volume. A few days later, the patient complained of a generalized itch and jaundice. Laboratory tests highlighted hematocrit 31,2%, platelets 636.000/ml, increased liver enzymes (AST 3,2 ukat/l, ALT 9,55 ukat/l), total bilirubin 7,9 umol/L, increased bile acids 48 umol/L. Viral hepatology tests (hepatitis A, B and C) were negative. Hepatic ultrasound scan showed a slightly inhomogeneous liver structure, dense bile and non-dilated bile ducts. Gastroenterologic advice diagnosed advanced ICP following OHSS. The patient started a treatment with ursodeoxycholic acid 300, mg twice a day, which brought to an improvement of her clinical conditions. A new ultrasound scan showed two gestational sacs, one containing a fetus at 8 weeks of gestational age and the other without embryo. Free fluid was absent and ovaries presented a regular volume. Laboratory analysis showed that AST, ALT and bile acid levels were 0,42 ukat/l, 2,23 ukat/l, and 4,1 umol/L respectively, and reached normal levels in one week, so the patient was discharged. Blood tests performed during pregnancy and after delivery showed a normal liver function. The pregnancy continued without other complications until the 36^th^ week of gestation, when the patient developed ICP in the third trimester, becoming symptomatic (itch). The patient underwent cesarean section because of increased levels of bile acids (86 umol/L) and acute fetal distress during cardiotocographic (CTG) monitoring; the newborn was a healthy baby of 2500 g with an Apgar score of 9–10. Both the post-partum and the puerperium periods had a regular course. Based on a multidisciplinary discussion with gynecologists, anesthesiologists, gastroenterologists and a molecular biologist expert in genetics predisposition, a genetic study was performed on the patient analyzing the possible presence of multi-drug-resistance protein 3 (*MDR3*) gene variants in exons 14, 15 and 16, but the sequence analysis of the polymorphisms resulted negative.

Our report follows the Consensus-based Clinical Case Reporting (CARE) Guideline Development [10], validated by the Enhancing the QUAlity and Transparency Of health Research (EQUATOR) network. The patient signed informed consent for the procedure she underwent, to allow data collection for research purpose and the publication of this case report. Considering the anonymized data collection and description of the case, a formal Institutional Review Board approval was exempted.

## Discussion and conclusions

ICP is a relatively uncommon disorder that usually appears during the third trimester of gestation, and it usually resolves within a few days from the delivery [1, 11]. This condition has also been observed rarely in the first trimester [2]. Although probably multifactorial, the exact pathogenesis of ICP is unknown and many theories regarding it have been proposed [11–14]. Specific risk factors for ICP include multiple gestation [15] and a previous history of itch while on oral contraceptive pills [15]. These conditions are related to a hyperestrogenism status [15] or to estrogenic compounds [16]. Estrogens reduce bile flow through Na–K-ATPase activity suppression [17] and they increase biliary cholesterol secretion [18]. OHSS is characterized by supraphysiological levels of estrogens and abnormalities in liver function parameters [19] and, as a consequence, liver dysfunctions have been reported in 30–40% of patients with OHSS following IVF [20]. Furthermore, the circulatory dysfunctions and the increased vascular permeability that characterize severe OHSS may also lead to liver dysfunction [21, 22]. Indeed, both microvascular thromboses and increased permeability of the hepatic vasculature may also lead to hepatocellular damage resulting in an increment of AST and ALT [21, 22]. Liver biopsies performed in OHSS patients have also showed ultrastructural changes consistent with increased hepatic enzyme activity, such as mitochondrial crystalline inclusions and dilatation of the rough endoplasmic reticulum [23].

Moreover, mutations in the hepatocellular phospholipid transporter ABCB4 (MDR3), and in the bile acid export pump ABCB11/BSEP, seem to be predisposing factors for developing ICP [14, 24, 25]. Indeed, *MDR3* is an ABC transporter (ABCB 4) that acts as a phospholipids flippase required for the biliary excretion of phosphatidylcholine [26] and its mutations have been found to have an important role in adult cholestatic syndromes [14] and to be present in 15% of all ICP cases [25, 26].

The temporary liver dysfunction that can occur during OHSS is reversible and it improves with decreasing estrogen levels [20, 22]. In this paper we describe a case of first trimester ICP that occurred after OHSS, and a literature review was performed with the aim of identifying all other case reports of ICP in the first trimester of pregnancy after OHSS. The literature search was performed using PubMed, Scopus, and Web of Science, without language restrictions, including publications until February 2022. Search terms included “intrahepatic cholestasis of pregnancy” in combination with “liver dysfunction”, “first trimester”, “ovarian hyperstimulation syndrome” and “IVF”. To the best of our knowledge, all reported cases and available data are summarized in Table 1. Considering only manuscripts reporting ICP in the first trimester after OHSS, we found ten reports published [3, 22, 27–34]. Two of the articles reported two cases [3, 28]. Including our case, all reported pregnancies obtained with ARTs were complicated by OHSS. Four cases were twin pregnancies [3, 28, 34] and one was a triplet pregnancy [29]. All cases of first trimester ICP occurred after moderate to critical OHSS and the liver dysfunction appeared between the 6^th^ and the 9^th^ week of gestation.

**Table 1:** Detailed presentation of all available cases of intrahepatic cholestasis in the first trimester of pregnancy after ovarian hyperstimulation syndrome

Authors and year	Type of pregnancy	OHSS	Gestational age at diagnosis	Liver function tests	ICP in the 3^rd^ trimester	Delivery
Ryley et al. 1990 [23]	N/A	Severe	7 weeks	• Bile acid N/A • AST 255	—	Early miscarriage
Shimono et al. 1998 [27]	Singleton	Moderate	7 weeks	• Bile acid 172.5 uM/L • ALT 3.2 ukat/l • AST 5.4 ukat/l	—	Early miscarriage
Midgley et al. 1999 [28]	Twin	Moderate	9 weeks	• Bile acid 166 uM/L • ALT 4.17 ukal/l • AST N/A	Yes	Cesarean section at 34^+2^ weeks gestation for abnormal umbilical artery Doppler of one twin
Elter et al. 2001 [29]	Triplet	Moderate	6 weeks	• Bile acid N/A • ALT 4.4 ukat/l • AST 7.6 ukat/l	N/A	N/A (ongoing pregnancy when the case was reported)
Davis et al. 2002 [30]	Singleton	Severe	6 weeks	• Bile acid N/A • ALT 7.7 ukat/l • AST 7.5 ukat/l	—	Blighted ovum
Wanggren et al. 2004 [31]	Singleton	Moderate	7 weeks	• Bile acid N/A • ALT 1,87 ukat/l • AST 2,98 ukat/l	Yes	Vaginal delivery at term
Obrzut et al. 2005 [32]	Singleton	Critical	7 weeks	• Bile acid N/A • ALT 56.2 ukat/l • AST 19.3 ukat/L	No	Vaginal delivery at term
Zamah et al. 2008 [33]	Singleton	Severe	7 weeks	• Bile acid 137.2 uM/L • ALT 2.75 ukat/l • AST 7.08 ukat/l	No	Vaginal delivery at term
	Singleton	Moderate	6 weeks	• Bile acid 308 uM/L • ALT 2.27 ukat/l • AST 2.05 ukat/l	—	Early miscarriage
Sarikaya et al. 2012 [34]	Twin	Critical	8 weeks	• Bile acid N/A • ALT 5.33 ukat/l • AST 3.17 ukat/l	No	Cesarean section at 34 weeks of gestation for hypertension
Mutlu et al. 2017 [3]	Singleton	moderate	6 weeks	• bile acid 166.8 uM/L • ALT 15.1 ukat/l • AST 6.67 ukat/l	—	Blighted ovum
	Twin	Severe	6 weeks	• Bile acid 122 uM/L • ALT 2.29 ukat/l • AST 1.80 ukat/l	No	Cesarean section at 36^+2^ weeks of gestation due to spontaneous membrane rupture
Our case	Twin	Critical	7 weeks	• Bile acids 48 umol/L • ALT 9,55 ukat/l • AST 3,2 ukat/l	Yes	Blighted ovum; caesarean section for increased bile acids levels and CTG pathologic alterations at 36 weeks of gestation

*OHSS* Ovarian hyperstimulation syndrome, *ICP* intrahepatic cholestasis of pregnancy, *AST* Aspartate aminotransferase, *ALT* Alanine aminotransferase, *N/A* Not available, *CTG* Cardiotocography

With the onset of the pregnancy, the massive corpora lutea that developed during the COH complicated by OHSS are rescued [35] and they supply high circulating levels of sex steroids during the first trimester [36–39]. Therefore, hormone levels are high in all reported pregnancies, and this could explain the mechanism underlying the development of ICP in the first trimester. Moreover, serum estrogen levels increase with the onset of a multiple pregnancy [40]. Why only few patients among women who achieved a pregnancy after moderate or severe OHSS present ICP remains unclear. One reason could be genetic predisposition, since genetic factors represent a concurrent factor for ICP development [23–25]. To the best of our knowledge, we report the first case of ICP developed in the first trimester of pregnancy after OHSS in which genetic polymorphisms of ABCB4 (MDR3) have been investigated. In our case, the sequence analysis of the polymorphisms resulted negative, confirming that the MDR3 mutations could be considered responsible for the development of ICP in only in a small percentage of Italian women [24]. We take the opportunity to acknowledge that investigation of genetic polymorphisms of ABCB4 is not routinely performed in our center: the decision to perform this type of genetic test was done after multidisciplinary discussion of the case, considering the unusual development of ICP during the first trimester in a pregnancy after OHSS, which further amplified the complexity of the case. Nevertheless, we fairly highlight that the potential advantage of this genetic investigation is minimal, aimed to identify patients which are at higher risk of developing ICP compared to general population; in addition, once the diagnosis of ICP is done, the management strategy would be not affected by the genetic status of the patients. From this perspective, we remark that screening for genetic polymorphisms of ABCB4 on the overall pregnant population could not be considered cost-effective, nor evidence-based.

In the reported cases, including our case, seven women were able to deliver [3, 27, 28, 30–34], and for five women the pregnancy outcomes were unfavourable [3, 22, 27, 30, 33]. Among the women who delivered, three delivered at term [31–33] and one of them developed ICP during the third trimester [31]. All the other women delivered prematurely through caesarean section [3, 28, 34]: two of them developed ICP during the third trimester [28] and one developed hypertension [34] (Table 1). In our case, caesarean section was performed at the 36^th^ week of gestation because of the increased bile acids levels and pathologic alterations that emerged during CTG monitoring. We take the opportunity to remark that more than half of the cases (6/10) we found in the literature did not report total bile acid level (Table 1). Since elevated total bile acid level is a pre-requisite for delivery, this may be considered a significant bias to be considered for a proper data interpretation.

Despite the small number of reported cases, it seems that ICP recurrence during third trimester might be possible when it has already been developed during the first trimester, probably because during the third trimester estrogen levels rise again [37] and these women have a predisposition for ICP development [38]. ICP is associated with adverse pregnancy and perinatal outcomes such as preterm labor, fetal asphyxia, meconium-stained amniotic fluid, and stillbirth and with an increased risk of preeclampsia, especially among women with an early ICP manifestation and twin gestations [39]. Therefore, it would be wise to monitor for the biochemical levels of liver enzymes, bilirubin and bile acids during the third trimester in women who developed ICP in the first trimester, since cholestasis can be asymptomatic and itch can either not be present as an alarm symptom or it can appear after the liver dysfunction [40]. In these women it might also be useful to check for *ABCB4 (MDR3)* and *ABCB11/BSEP 3* gene polymorphisms to know if they have a predisposition for ICP recurrence during the third trimester or in other conditions characterized by hyperestrogenism. It could also be interesting to investigate if the ICP during the first trimester can be harmful for the developing embryo.

## Data Availability

All data generated or analysed during this study are included in this published article.

## Abbreviations

ICP: Intrahepatic Cholestasis of Pregnancy
COH: Controlled Ovarian Hyperstimulation
OHSS: Ovarian Hyperstimulation Syndrome
CTG: Cardiotocography
ARTs: Assisted Reproduction Technologies
IVF: In Vitro Fertilization
GnRH-a: Gonadotropin Releasing Hormone agonist
rFSH: Recombinant Follicle-Stimulating Hormone
rhCG: Recombinant human Chorionic Gonadotrophin
